# Electrochemistry-mass spectrometry for mechanism study of oxygen reduction at water/oil interface

**DOI:** 10.1038/srep46669

**Published:** 2017-04-24

**Authors:** Shu-Juan Liu, Zheng-Wei Yu, Liang Qiao, Bao-Hong Liu

**Affiliations:** 1 Department of Chemistry, State Key Laboratory of Molecular Engineering of Polymers and Institutes of Biomedical Sciences, Fudan University, Shanghai, 200433, China; 2 Shanghai Stomatological Hospital, Fudan University, Shanghai, 200001, China

## Abstract

Electrochemistry methods have been widely employed in the development of renewable energy, and involved in various processes, e.g. water splitting and oxygen reduction. Remarkable progress notwithstanding, there are still many challenges in further optimization of catalysts to achieve high performance. For this purpose, an in-depth understanding of reaction mechanism is needed. In this study, an electrochemistry-mass spectrometry method based on a Y-shaped dual-channel microchip as electrochemical cell and ionization device was demonstrated. Combined solutions of aqueous phase and oil phase were introduced into mass spectrometer directly when electrochemical reactions were happening to study the reduction of oxygen by decamethylferrocene or tetrathiafulvalene under the catalysis of a metal-free porphyrin, tetraphenylporphyrin, at water/1,2-dichloroethane interfaces. Monoprotonated and diprotonated tetraphenylporphyrin were detected by mass spectrometer, confirming the previously proposed mechanism of the oxygen reduction reaction. This work offers a new approach to study electrochemical reactions at liquid-liquid interface.

Today, fossil fuels are still primary energy sources accounting ~66% of the world total energy consumption, and their consumption is predicted to continue increasing as world economy and population growth^1^. Accelerated consumption of fossil fuels may cause severe economic, environmental, and geopolitical problems^2^. In this context, a transformation from fossil fuels to renewable energy, such as electrochemical energy^3^, is urgently needed to avoid depletion of fossil fuels. Among various strategies, electrochemistry (EC) at an interface between two immiscible electrolyte solutions (ITIES) has drawn considerable attention because of its outstanding performances in phase transfer catalysis^4^, solar energy conversion^5^, H_2_ and O_2_ evolution^6^, oxygen reduction^7^, etc. Remarkable progress notwithstanding, there are still many challenges in further optimization of catalysts to achieve high performance. To do so, an in-depth understanding of reaction mechanism is needed.

Mechanism study of electrochemical reactions at liquid-liquid (L/L) interfaces is usually carried out using cyclic voltammetry^8^, spectroscopy^9^, scanning electrochemical microscopy (SECM)^10^, scanning ion conductance microscopy (SICM)^11^, etc. However, these methods lack high chemical specificity. In this regard, mass spectrometry (MS) can serve as a sensitive detector to identify products and intermediates generated during electrochemical processes by providing molecular weight and fragments information^12^.

EC coupled to MS (EC/MS) was first introduced by Bruckenstein *et al*. in 1971^13^. Since then, EC/MS has been a powerful tool to study EC reactions^14^,^15^. Zare’s group utilized a rotating waterwheel electrode cell coupled with desorption electrospray ionization (DESI) to detect transient intermediates generated on electrode surface for mechanism study^16^,^17^. Pastor *et al*. developed an electrochemical MS configuration to study ethanol electro-oxidation in acidic medium^18^. To date, most work focused on EC reactions on electrode surface or in a single phase. As one of the first attempts to study EC at L/L interfaces, Hubert Girault *et al*. have investigated nucleophilic substitution on ferrocene methanol^19^, different interfacial complexations, e.g. the complexation of lead ions by thioether crown molecules, dipeptide by dibenzo-18-crown-6^20^, and peptides by phospholipid^21^, by biphasic electrospray ionization (ESI)-MS. However, MS detection of intermediates generated by EC reactions at L/L interface still needs to be further explored.

In this work, we use a Y-shaped dual-channel microchip as an electrochemical cell and an ionization device to couple with MS to monitor oxygen reduction reactions (ORR) at L/L interfaces. ORR is of utmost importance in advanced electrochemical energy conversion^22^. ORR at L/L interfaces has been widely studied using various lipophilic electron donors, e.g., ferrocene (Fc), DMFc, TTF, etc^7^,^23^,^24^,^25^,^26^. Compared to other complicated reaction systems, ORR at water/1,2-dichloroethane (DCE) interfaces catalyzed by metal-free porphyrin, tetraphenylporphyrin (H_2_TPP), has been explored thoroughly^25^,^26^. The possible mechanism of two consecutive protonations of H_2_TPP leading to the coordination of porphyrin diacid by molecular O_2_ and reduction of oxygen was proposed and supported by cyclic voltammetry, UV-visible spectrometry, and model calculation^23^,^25^. Herein, with the assistance of the fine-designed microchip, ORR at water/DCE interfaces catalyzed by H_2_TPP were carried out in microchannels, and the L/L interface was delivered into mass spectrometer during the happening of electrochemical reactions. The EC was carried out either under the application of external voltage, or in the presence of lithium tetrakis(pentafluorophenyl) borate (LiTB) in aqueous solution without external voltage. Key intermediates, such as monoacid H_3_TPP^+^ and diacid H_4_TPP^2+^, were observed, directly demonstrating the proposed mechanism in literature^23^,^25^.

## Results and Discussion

### EC-MS study of ORR by TTF at L/L interface controlled by LiTB

**Figure 1:**
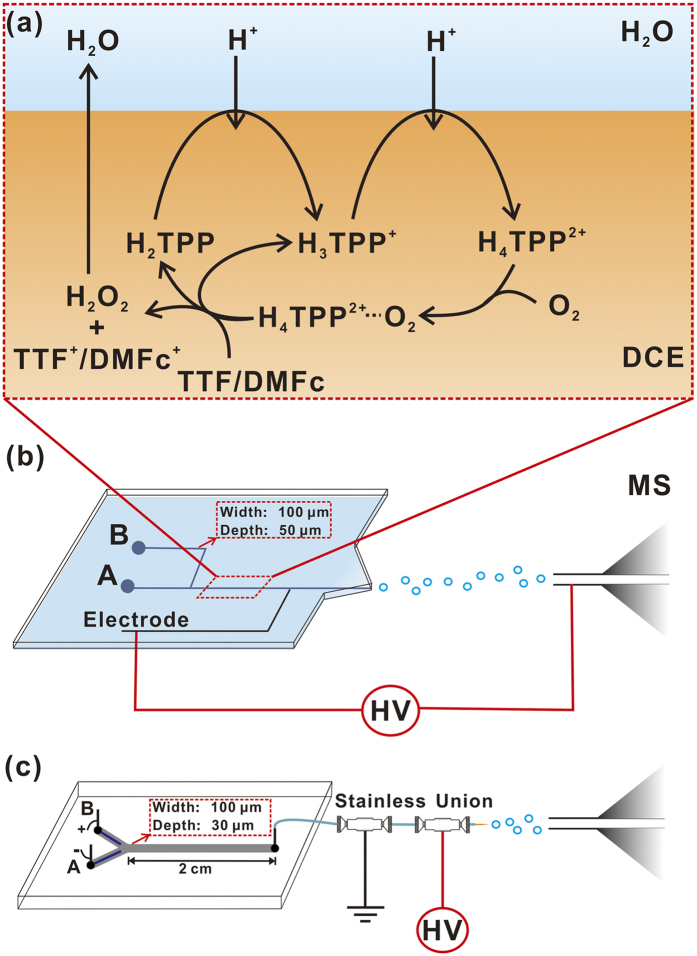
(**a**) Proposed mechanism of oxygen reduction by DMFc or TTF catalyzed by H_2_TPP at water/DCE interfaces. Microchip coupled MS to characterize biphasic reaction products (**b**) without and (**c**) with external potential for EC reactions. Channel A was used for organic solution, and channel B for aqueous solution.

ORR at soft interfaces can be controlled by the addition of LiTB into an acidic aqueous phase^4^. In this way, the reaction can be carried out by simply infusing aqueous solution and oil solution into the microchip as shown in Fig. 1b, which is also an emitter of ESI-MS. An aqueous phase containing LiTB and H_2_SO_4_ was introduced to channel A and DCE containing tetramethylammonium tetrakis(pentafluorophenyl) borate (TMATB), DMFc/TTF and H_2_TPP was introduced to channel B. The compositions of electrochemical cells are depicted by Cells 1 and 2.

Cell 1: 5 mM LiTB + 5 mM H_2_SO_4_(w)||5 mM TMATB + 5 mM TTF + 20 mM H_2_TPP (DCE)

Cell 2: 5 mM LiTB + 5 mM H_2_SO_4_(w)||5 mM TMATB + 5 mM DMFc + 20 mM H_2_TPP (DCE)

Cell 3: 10 mM LiCl + 100 mM HCl(w)||5 mM BTPPATPBCl + y mM TTF + x mM H_2_TPP (DCE)

Cell 4: 10 mM LiCl + 100 mM HCl (w)||5 mM BTPPATPBCl + 5 mM DMFc + 20 mM H_2_TPP (DCE)

**Figure 2:**
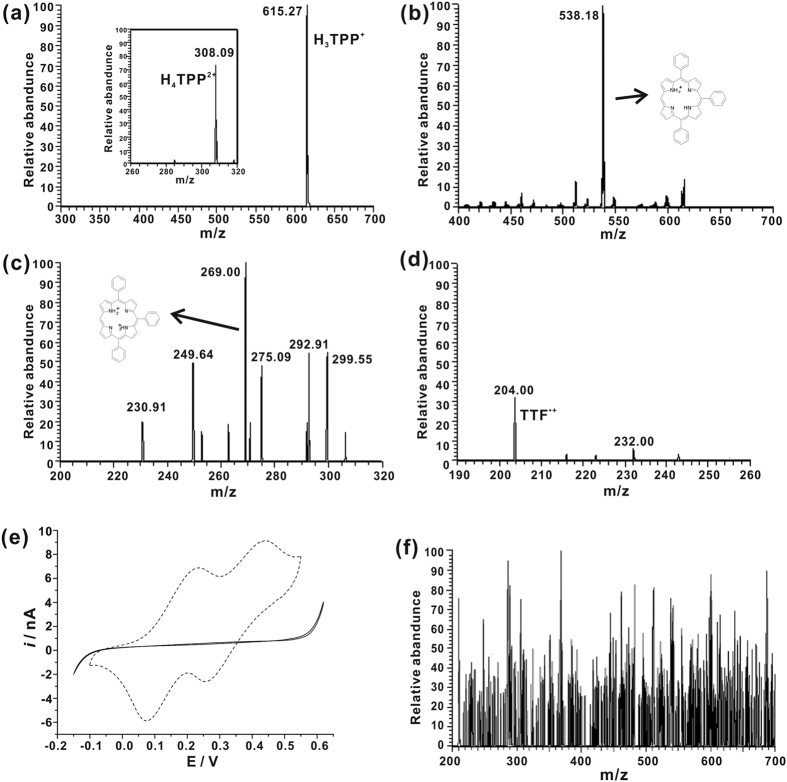
(**a**) Mass spectrum of the reaction system in cell 1: 5 mM TTF, 5 mM TMATPFB, and 20 mM H_2_TPP in DCE infused *via* channel A; 5 mM LiTB in 5 mM H_2_SO_4_ solution infused *via* channel B. The inset shows the peak of porphyrin diacid H_4_TPP^2+^. (**b**) CID of parent ion at m/z = 615. (**c**) CID of parent ion at m/z = 308. (**d**) Mass spectrum with m/z range of 190 to 260 of the reaction system in cell 1. (**e**) Cyclic voltammograms obtained with electrochemical cell 3: x = 0 and y = 0 (solid line); x = 5 and y = 20 (dashed line). The scan rate was 25 mV/s. *r* = 1 μm. (**f**) Mass spectra of the reaction system in cell 1 without LiTB: 5 mM TTF, 5 mM TMATPFB, and 20 mM H_2_TPP in DCE infused *via* channel A; 50% water, 49% methanol and 1% acetic acid infused *via* channel B.

ORR by TTF at water/DCE interfaces was firstly tested. According to previous publications, ORR catalyzed by H_2_TPP involves two consecutive protonations of H_2_TPP to form monoacid H_3_TPP^+^ and diacid H_4_TPP^2+^, leading to the coordination of porphyrin diacid by molecular O_2_ and reduction of oxygen (Fig. 1a)^25^,^26^. In the mass spectrum of TTF reaction system (Fig. 2a), a significant peak at m/z 615 was observed, indicating the generation of H_3_TPP^+^ in the microchip. Furthermore, the ion was fragmented by collision-induced dissociation (CID). A predominant fragment ion at m/z 538 was produced, corresponding to the loss of a phenyl group from the ion with m/z 615, further confirming that the peak at m/z 615 corresponded to H_3_TPP^+^ (Fig. 2b). It was hard to observe the peak of H_4_TPP^2+^ by full MS scan mode. When we applied single ion monitor (SIM) mode with center m/z = 280, and range of 80 m/z, a peak at m/z 308 was observed, which was ascribed to H_4_TPP^2+^. By applying CID on the ion, a fragment ion at m/z 269 was observed, corresponding to the loss of a phenyl group from the ion with m/z 308 (Fig. 2c). The observation of both H_3_TPP^+^ and H_4_TPP^2+^ demonstrated the proposed mechanism shown in Fig. 1a. From the mass spectra, the peak intensity of H_3_TPP^+^ was much stronger than that of H_4_TPP^2+^, indicating a very short lifetime or low equilibrium concentration of H_4_TPP^2+^, which inferred that the rate-determining step is the coordination of porphyrin diacid with molecular O_2_. During the ORR process, TTF was oxidized to TTF^•+^, which was observed at m/z 204 (Fig. 2d), further demonstrating that ORR was taking place in the microchip. However, we did not observe any peaks for H_4_TPP^2+^-O_2_. The intermediate may suffer from a too-low stability or too short lifetime to be detected. In a control experiment, the organic phase of 5 mM TTF, 5 mM TMATPFB and 20 mM H_2_TPP in DCE, and the aqueous phase of 50% water, 49% methanol and 1% acetic acid were infused into the microchip as shown in Fig. 1b and analyzed by ESI-MS. No peak was observed for H_3_TPP^+^ or H_4_TPP^2+^ (Fig. 2f), demonstrating that H_3_TPP^+^ and H_4_TPP^2+^ were not simply from protonation of H_2_TPP in a mixture of aqueous and organic phase. H_3_TPP^+^ and H_4_TPP^2+^ can only form in the presence of LiTB or under the voltage for electrochemistry reactions.

The EC measurements of oxygen reduction by TTF in the presence of H_2_TPP were performed by cyclic voltammetry using electrochemical cell 3. A glass micropipette was employed, where water phase was filled into the pipette, and the pipette was put in DCE. Compared to blank response, two current waves at 0.23 V and 0.45 V were observed (Fig. 2e). According to previous publication, the first wave could be ascribed to the transfer of a proton from water to DCE facilitated by H_2_TPP, corresponding to the first protonation of H_2_TPP. The second one arose from the facilitated transfer of the second proton by H_3_TPP^+^ to form the diacid H_4_TPP^2+ ^25. The CV results corresponded well with the mass spectra in Fig. 2a.

**Figure 3:**
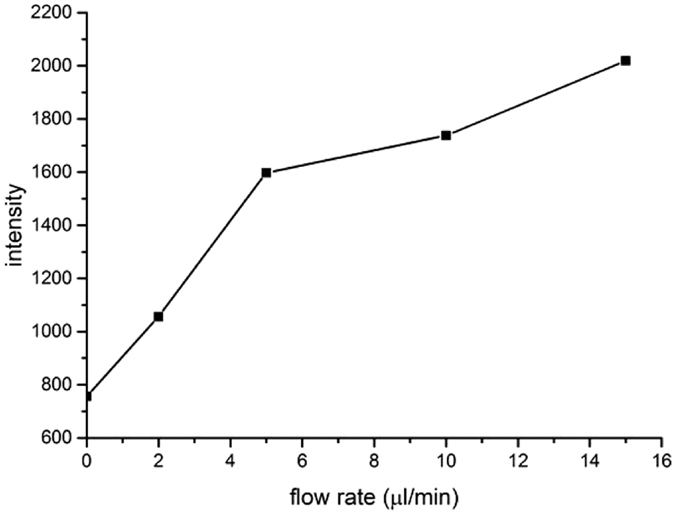
Dependence of signal intensity of the peak at m/z = 615 on sample infusion flow rates.

The influence of sample infusion flow rates in the microchip to signal intensity has also been investigated. The flow rates of aqueous solution and organic solution were the same and changed together. Figure 3 shows a strong positive correlation between the signal intensity of m/z 615 and the flow rates in the range of 0 to 5 μl/min, and a relatively weak positive correlation between the signal intensity and the flow rates in the range of 5 to 15 μl/min. With higher flow rates, more solutions could be transferred to the mass spectrometer at the same time interval. However, high flow rates also led to short reaction time. At very high flow rate, the production of H_3_TPP^+^ was limited because of short reaction time. Therefore, the signal intensity of H_3_TPP^+^ did not increase significantly. Limited by the microchip ESI, a combined flow rate >30 μl/min was not feasible. Without sheath gas flow, droplets could be accumulated at the end of the emitter to stop ESI when the combined flow rate is larger than 30 μl/min.

### EC-MS study of ORR by TTF at L/L interface controlled by external potential

**Figure 4:**
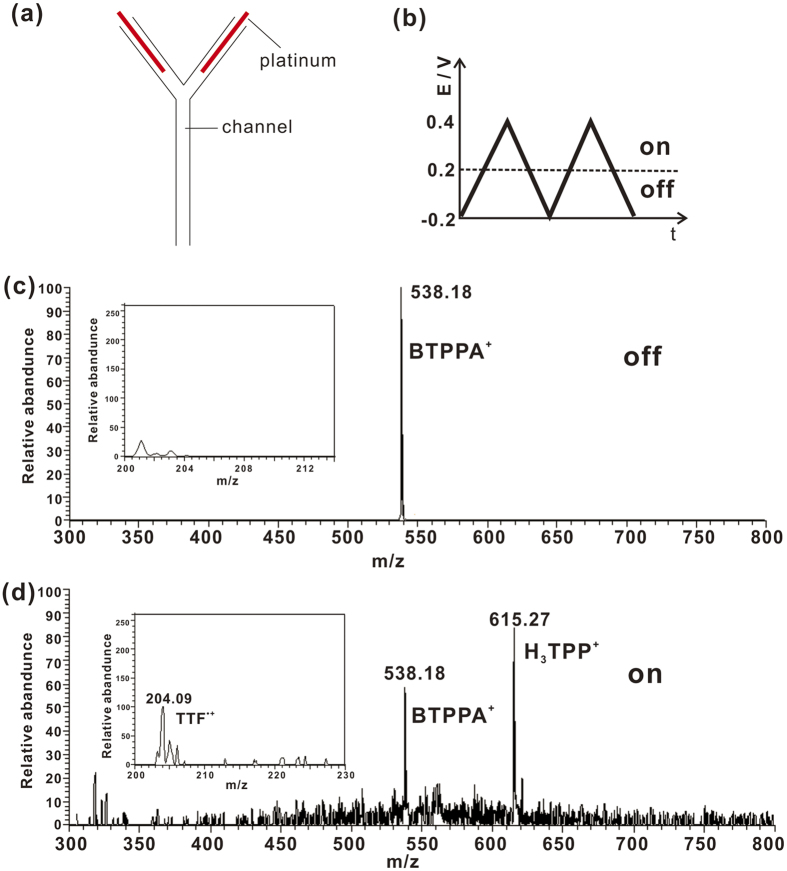
(**a**) Scheme of microchip channel with platinum electrode inside. (**b**) The external potential program applied on the microchip. (**c**) Mass spectra of the reaction system of cell 3 when the potential was below 0.2 V. The inset showed the scan result from m/z 200 to m/z 214. No effective signal of TTF^•+^ was observed. (**d**) Mass spectra of the reaction system of cell 3 when the potential reached 0.2 V. The inset showed the peak of TTF^•+^. Cell 3: x = 5 and y = 20. The scan rate was 10 mV/s.

To further prove the vitality of the protocol in combining EC with MS, we performed the electrochemical experiment of cell 3 using an improved microchip to couple with MS (Fig. 1c). The whole microchip was made of glass with a “Y” shaped channel. Two platinum lines were electroplated in the channel, serving as electrodes. External potential was applied though the two electrodes. Reaction products were directed from the outlet of the “Y” shaped channel into a mass spectrometer *via* ESI by using a pulled glass capillary as emitter. A metal valve was added between the electrochemistry microchip and the glass ESI emitter, as shown in Fig. 1c. The metal valve was grounded to decouple the high voltage for ESI and the voltage for EC. The tubing between the grounded metal valve and the metal valve with ESI high voltage was long enough to give sufficient resistance until a stable ESI could be observed. ORR could only happen when certain external potential was applied on the electrodes of the microchip. With the assistance of the improved microchip, we monitored the reaction process of cell 3 using MS while the potentiostat for EC was working. A CV program was applied on the microchip (Fig. 4b). When the potential was below 0.2 V, no ORR happened. Only the peak of electrolyte BTPPA^+^ at m/z 538 was observed, Fig. 4c. When the potential reached 0.2 V, the peaks of H_3_TPP^+^ at m/z 615 and TTF^•+^ at m/z 204 were detected, indicating the beginning of ORR (Fig. 4d). The result corresponds well with the EC experimental results in Fig. 2e.

### EC-MS study of ORR by DMFc at L/L interface

**Figure 5:**
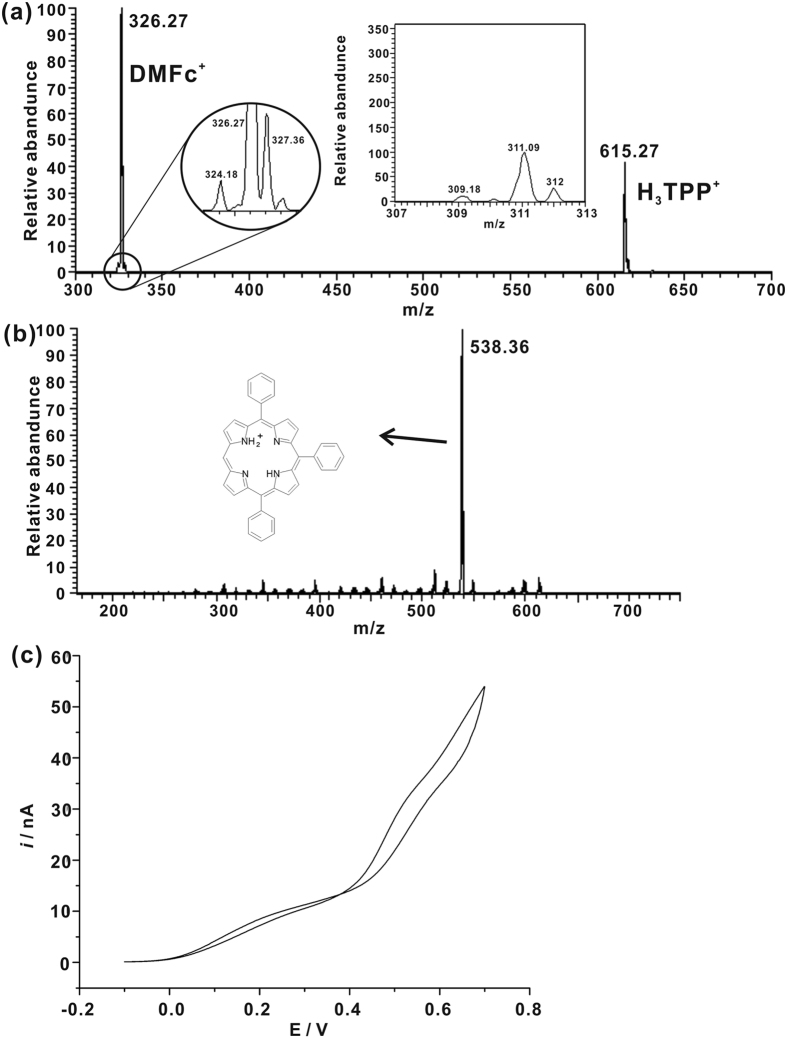
(**a**) Mass spectrum of the reaction system in cell 2. 5 mM DMFc, 5 mM TMATPFB, and 20 mM H_2_TPP in DCE infused *via* channel A, 5 mM LiTPFB in 5 mM H_2_SO_4_ solution infused *via* channel B. The inset shows the SIM scan result from m/z 307 to m/z 313. No effective signal of H_4_TPP^2+^ was observed. The circle shows the amplification of the peak at m/z 326. (**b**) CID of parent ion at m/z = 615. (**c**) Cyclic voltammograms obtained with electrochemical cell 4 with the scan rate = 25 mV/s, and *r* = 1 μm.

We have also studied ORR by DMFc (cell 2) with the microchip shown as Fig. 1b. The peak at m/z 615 was still well observed and confirmed by CID (Fig. 5), indicating the formation of mono-protonated H_2_TPP. The peak at m/z 326 was ascribed to the protonated DMFc. The circle in Fig. 5a shows the amplification of the peak at m/z 326. A peak at m/z 327 was indeed observed, which can be the isotope peak of DMFc^+^, or from a mixture of DMFc-H^+^ and DMFc^+^. According to abundance calculation, the relative intensity of the isotope peak of DMFc^+^ at m/z 327 should be 18.3% when the monoisotopic peak of DMFc^+^ at m/z 326 = 100%. In Fig. 5a, the relative intensity of the peak at m/z 327 was about 28.6%, while the relative intensity of the peak at m/z 326 was 100%, indicating that the peak at m/z 327.3 was from both DMFc-H^+^ and DMFc^+^. The observation of DMFc-H^+^ supported the mechanism proposed by Su *et al*.^27^ However, it was barely hard to detect the peak of H_4_TPP^2+^, indicating that the amount of H_4_TPP^2+^ in the reaction system was rather low. According to previous study^28^, ferrocenes, such as DMFc, can reduce O_2_ to generate H_2_O_2_ via a two-electron reduction pathway without catalyst. Compared to DMFc, TTF can only reduce O_2_ effectively in the presence of catalyst, such as metal-free porphyrins. When H_2_TPP was employed, the diacid intermediate, H_4_TPP^2+^, would combine with O_2_, and then reacted with DMFc or TTF to produce H_2_O_2_. Under the same experiment conditions, less H_4_TPP^2+^ was detected in the DMFc system than the TTF system, which inferred that H_4_TPP^2+^ was quickly consumed in the presence of DMFc, in accordance with the stronger electron-donating ability of DMFc than TTF. The EC measurements of oxygen reduction by DMFc in the presence of H_2_TPP (cell 4) were also performed as shown in Fig. 5c. Two current waves were observed, which can be ascribed to the formation of H_3_TPP^+^ and H_4_TPP^2+^, respectively.

## Conclusion

In summary, we report a microchip based EC/MS protocol to monitor electrochemical reactions at L/L interface. Microchips with Y-shaped channels were used as electrochemical cells. The combined solutions of aqueous phase and oil phase at the interface were induced to the inlet of MS directly when the electrochemical reactions were happening. ORR by DMFc/TTF in the presence of H_2_TPP at water/DCE interface was studied. The monoacid and diacid intermediates were detected to confirm the proposed mechanism of ORR. This work offers a new approach to study electrochemical reactions at L/L interface and to explore reaction mechanism.

## Methods

### Chemicals, reagents and materials

Sulfuric acid (H_2_SO_4_, AR) and hydrochloric acid (HCl, AR) were purchased from Zhitang Chemical Company. Tetramethylammonium chloride (TMACl, ≥98%, TCI), lithium tetrakis(pentafluorophenyl)borate ethyl etherate (LiTPFB, Aldrich), potassium tetrakis(4-chlorophenyl)-borate (KTPBCl, ≥98%, Aldrich), bis(triphenylphosphoranylidene)-ammonium chloride (BTPPACl, ≥98%, Aldrich), bis(pentamethylcyclopentadienyl)iron (DMFc, ≥97%, Aldrich), tetraphenylporphyrin (H_2_TPP, ≥98%, TCI), tetrathiafulvalene (TTF, ≥97%, Aldrich) were used as received without further purification. 1,2-Dichloroethane (DCE, ≥99%, Sinopharm Chemical Reagent Co.) was washed with triply distilled water before use. Bis-(triphenylphosphoranylidene)-ammonium tetrakis(4-chlorophenyl) borate (BTPPATPBCl) was synthesized by metathesis of equimolar solutions of BTPPACl and KTPBCl. The salts were recrystallized from acetone and then dried in an oven at 95 °C for 24 h. Tetramethylammonium tetrakis(pentafluorophenyl)borate (TMATPFB) was synthesized by the same method. All aqueous solutions were prepared from triply distilled water.

### Microchip preparation

A polyimide (PI) substrate (125 μm in thickness) based microchip was prepared by laser ablation. Two channels, A and B, with depth of 50 μm and width of 100 μm, were designed for liquid injection. The two channels joined together as shown in Fig. 1b to form a channel with depth of 50 μm, width of 100 μm and length of 3 cm, for on-chip L/L interface reactions. At the end of the channel, high voltage for ESI was applied *via* an electrode channel with depth of 30 μm and width of 100 μm filled with carbon paste. The PI substrate was laminated with a 50 μm PE composite sheets, where PE acted as the sealing agent when rolled at 90 °C for 5 s. To insure good lamination, the entire structure was additionally cured for 1 h at 80 °C.

The glass microchip was prepared by means of wet etching. A “Y” shaped channel, with depth of 30 μm and width of 100 μm, was etched on one piece of glass. The part where two liquids join together has a length of 2 cm. Two platinum lines (width of 40 μm and length of 8 mm) were electroplated on the other piece of glass with certain pattern. Then the two pieces of glass were bonded. A key point is to make sure the platinum lines fitted in the scope of the channel.

### Microchip-MS instrumentation

All MS experiments were performed using a linear ion trap (LTQ) mass spectrometer (Thermo Fisher Scientific, San Jose, CA USA). No carrier gas was required. A plume of ions was generated by the application of a high voltage on the microchip as showed in Fig. 1. The tip of the microchip was about 2 mm away from the inlet of the mass spectrometer. Typically, the aqueous solution and oil were infused at a flow rate of 0–15 μl/min each. The MS was operated under the following parameters: microscans = 1, ion injection time = 60 ms, tube lens voltage = 205 V.

### EC measurements

Current−voltage curves were obtained with an electrochemical workstation (CHI, CH Instruments Ins.). Pipettes were fabricated by a CO_2_-laser-based pipet puller (P-2000, Sutter Instrument Co.) with quartz capillaries (0.7 mm I.D. and 1.0 mm O.D.). Aqueous solution was backfilled into the pipettes using a microfill needle, and then the pipettes were tapped to drive out air bubbles and checked under a microscope. An Ag/AgCl electrode was inserted in the pipet, and another Ag/AgCl reference electrode was placed in external organic bath.

## Additional Information

**How to cite this article**: Liu, S.-J. *et al*. Electrochemistry-mass spectrometry for mechanism study of oxygen reduction at water/oil interface. *Sci. Rep.* **7**, 46669; doi: 10.1038/srep46669 (2017).

**Publisher's note:** Springer Nature remains neutral with regard to jurisdictional claims in published maps and institutional affiliations.
